# Reduction of Operative Time, Intraoperative Radiographs, and Anterior Knee Pain With the Lateral Parapatellar Approach for Tibial Shaft Fracture Nailing: A Case Series

**DOI:** 10.7759/cureus.47309

**Published:** 2023-10-19

**Authors:** Deya AlWadi, Murad Jweinat, Ahmad Almigdad, Fadi AlRousan, Ahmad Alawamleh, Laith Hseinat

**Affiliations:** 1 Department of Orthopaedics, Jordanian Royal Medical Services, Amman, JOR

**Keywords:** decreased operative time, semi-extended, anterior knee pain, radiation dose reduction, tibia nail, tibia shaft fracture, lateral parapatellar

## Abstract

Background

Tibia fracture is a common indication for operative intervention in orthopedics. Usage of Intramedullary nailing provides a minimally invasive technique with good results. Positioning, operative time, and radiation exposure are major points in such cases. This study described the semi-extended lateral parapatellar intramedullary technique as a technique that helps achieve and maintain reduction, simplifies nail insertion, and decreases fluoroscopy and operative time.

Methodology

This prospective case series study included nine patients with tibial shaft fractures operated at Royal Rehabilitation Center from April to October 2023 by intramedullary nailing and extra-articular lateral parapatellar insertion technique using the semi-extended knee position. Duration of surgery, intraoperative radiation exposure, and anterior knee pain score were assessed for all patients.

Results

The average duration of surgery was 63.78 ± 5.3 minutes, and the average intraoperative imaging was 94 (85-103). The average union time was 18 ±2 weeks. The mean Kujala score was 90.9% ± 2.3% six months after the surgery. All patients regained a comparable range of motion in their knees and ankles. One patient reported minimal pain at the pin site but did not require any analgesia.

Conclusion

The lateral parapatellar approach for tibial shaft fracture nailing has the advantage of reducing operative time, the number of intraoperative radiographs, and lower postoperative anterior knee pain. Additionally, this approach did not cause patellar instability.

## Introduction

Tibia shaft fracture is one of the most common long bone fractures that orthopedic trauma surgeons deal with regularly [1,2]. However, tibia fracture treatments vary according to many factors, including patient age, fracture morphology, soft tissue status, experience of the surgeon, and the bioavailability of implants [3].

Intramedullary nailing is one of the most adopted fixation methods for tibia shaft fracture in adult patients. However, such minimally invasive procedures require high radiation exposure compared to other fixation methods [4]. Many operating theater workers are unaware of the amount of radiation exposure during surgery.

Anterior knee pain is the most common complication after the nailing of the tibial shaft fractures, mainly in active young adults [5-7]. Although the causes of anterior knee pain following tibia nailing were often discussed, the exact cause remains unclear. Many anatomical structures, including the infrapatellar fat pad, articular cartilage, and meniscus, are vulnerable to injury during the procedure. Additionally, mechanical causes such as the prominence of the nail, proximal screw position, and the possibility of proximal tibiofibular joint disruption may play a role [8-13].

During intramedullary tibia nailing, the leg is flexed to allow guide wire insertion and reaming. However, this position may adversely affect achieving and maintaining reduction during the procedure, leading to longer operative time and more fluoroscopy exposure [14]. Multiple surgical approaches regarding intramedullary nailing for tibia shaft fracture have been discussed, including infrapatellar, parapatellar, and suprapatellar approaches in varying knee positions [15,16].

In this study, we aim to confirm our hypothesis that using the semi-extended lateral parapatellar intramedullary technique may help achieve and maintain reduction, simplify nail insertion, and decrease fluoroscopy and operative time by designing a case series study to document our findings regarding anterior knee pain, operative time, and intraoperative radiation exposure.

## Materials and methods

We conducted a single-center prospective case series study on patients admitted with tibial shaft fractures to the orthopedic department at the Royal Rehabilitation Center at King Hussein Medical City in Amman, the capital of Jordan. Ethical approval was obtained through the ethical committee in the Royal Medical Services (number: 10/2023/12).

Inclusion criteria include adults above 18, isolated tibia shaft fracture, and closed injury. Exclusion criteria include ages under 18 years old, open fractures, multi-trauma patients, previous knee surgery or injury, patellar instability, and patients who were lost to follow-up during the study. Written patients' consents were obtained for the study.

We reviewed all the patients who were admitted and operated on in our center with tibia shaft fractures in March 2023, and the follow-up started roughly at the beginning of April 2024 and followed six months after. Nine patients met our criteria. All patients were treated for tibial shaft fracture with the semi-extended lateral parapatellar intramedullary tibia nailing. Patients were followed for six months to evaluate the postoperative outcome.

Collected data included age, gender, mechanism of injury, surgery duration, anterior knee pain scoring, and number of intraoperative fluoroscopic images. Intraoperative fluoroscopic radiation was represented by the number of static X-rays taken by an intraoperative c-arm. Patients were followed at six weeks, three months, and six months with leg radiographs to assess healing. At the final follow-up, clinical and radiological evaluation with knee and ankle range of movement was assessed. Additionally, anterior knee pain was measured using the Kujala anterior knee pain scoring system [17].

Statistical data analysis

The mean and standard deviation were used to describe the continuous variables and the frequencies and percentages for the categorical variables.

Surgical technique

**Figure 1:**
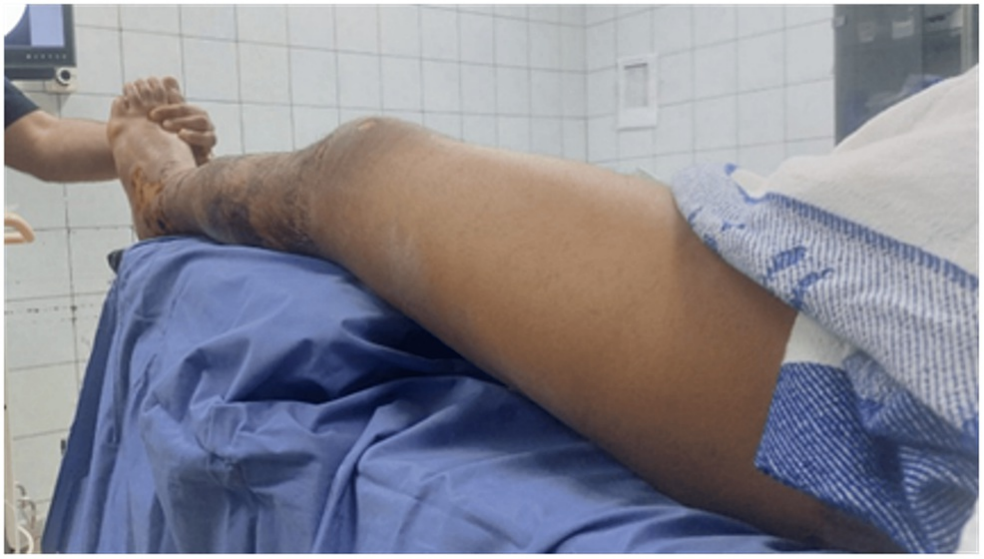
The intraoperative setting.

**Figure 2:**
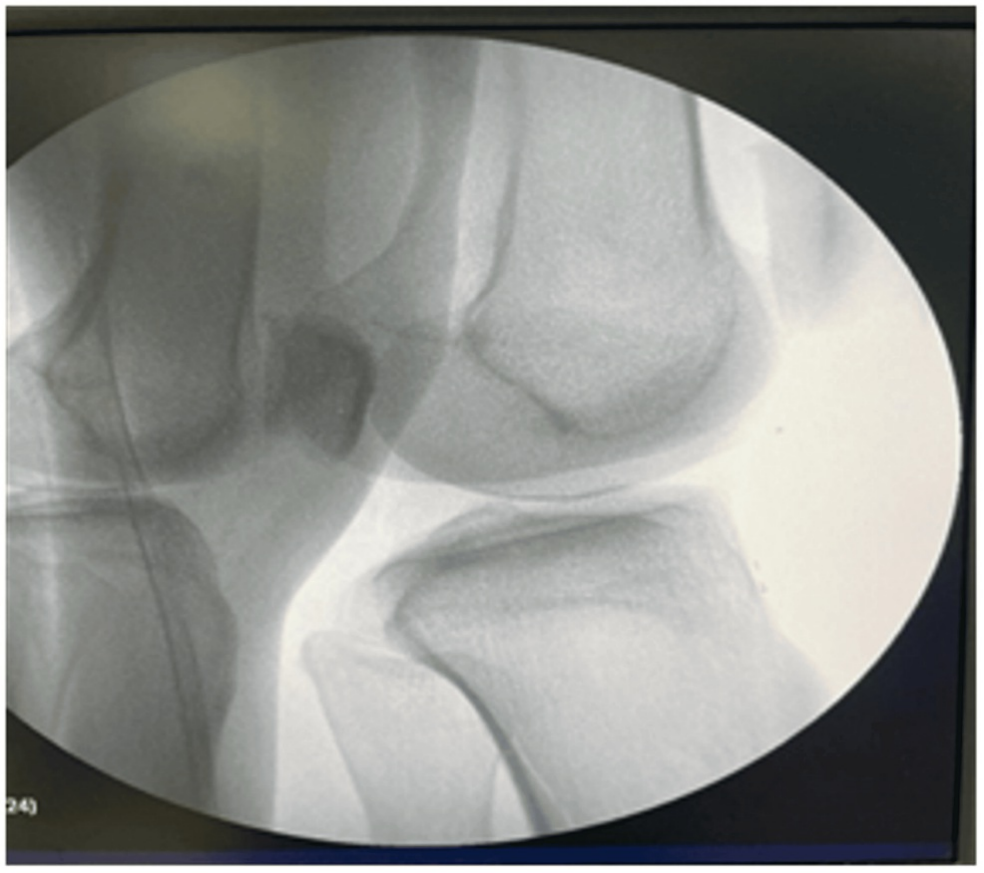
A lateral view without moving any of the limbs.

A preoperative assessment for patellar instability was done, which showed stable patella in all patients. Surgeries were performed under general or regional anesthesia. The patients were supine, and the injured leg was elevated using support underneath with 20-30 degrees of hip flexion to help take lateral X-rays and make the entry point more accessible (Figures 1-2).

**Figure 3:**
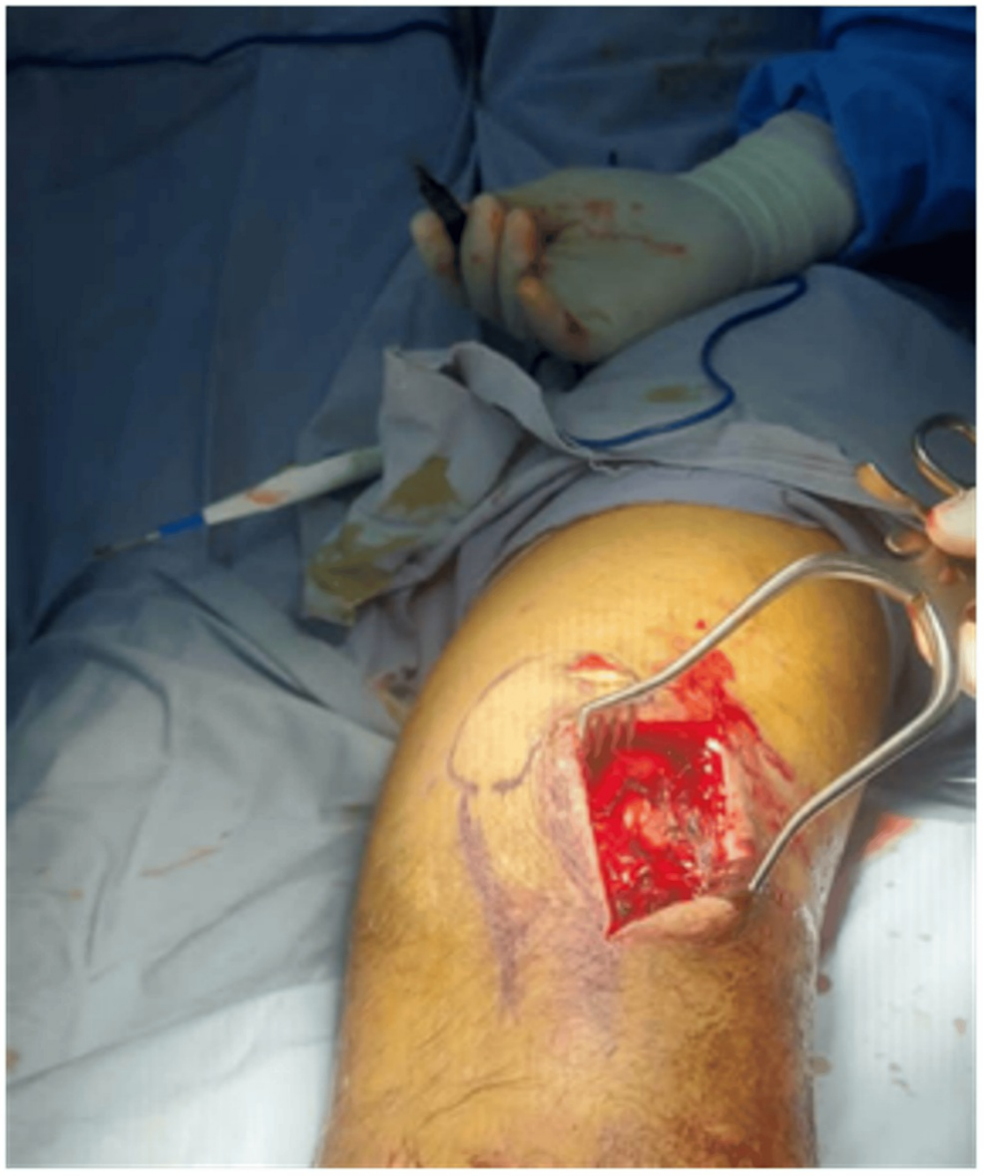
Skin anatomical surface markings.

Anatomical landmarks were placed, and a skin incision was marked lateral to the lateral border of the patellar in a curve shape inferiorly downward (if needed) and lateral to the patellar tendon (the convex side is lateral) (Figure 3).

Meticulous blunt dissection was done until it reached the lateral retinaculum. The lateral retinaculum was opened carefully, leaving 1.5-2 cm of the retinaculum attached to the patella, and dissection was conducted to the infrapatellar fat pad. Then, the fat pad and the patella were retracted medially. Using the fluoroscopic guidance, the entry point was identified using an awl, and the position was checked on anterior-posterior and lateral view.

**Figure 4:**
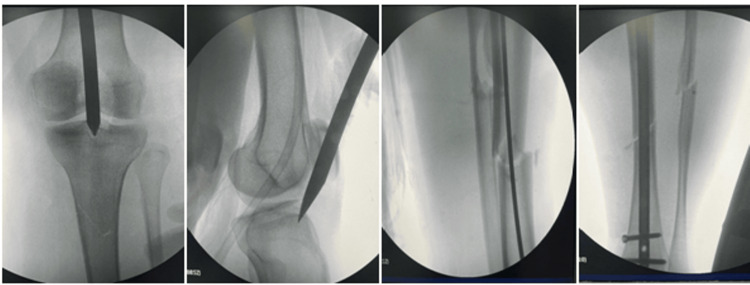
Intraoperative radiographs showing clear entry points and the introduced olive guide wire and nail in the semi-extended knee position. This patient had a proximal fracture line at the proximal tibia because of injury.

The guide wire was introduced, and then closed reduction was achieved in all cases where the guide wire was passed over the fracture site after that. A tissue protector was placed, and reaming with caution to the intra-articular tissue was done. Measures of the nail were taken, and then the nail was placed. Proximal and distal locking screws were placed as usual (Figure 4).

The retinaculum was repaired using Vicryl size 1 (Ethicon Inc., Somerville, NJ), and subcutaneous tissue and skin were closed. Patients were started on weight bearing as tolerated from the first day post-surgery.

## Results

The study included nine patients, comprising six (66.6%) males and three (33.3%) females, who all underwent surgery at the same center. The most common cause of injury was falling from the ground level, accounting for 88.9% of cases (Table 1).

**Table 1:** Descriptive analysis of the patients and the outcomes. RTA: Road traffic accident.

Case number	Gender	Age in years	Etiology	Number of intraoperative radiographs	Surgery duration (minute)	Knee pain score at six months
1	Male	27	Falling down	103	71	88%
2	Male	30	RTA	99	68	93%
3	Male	31	Falling down	93	70	92%
4	Female	38	Falling down	85	60	93%
5	Male	25	Falling down	95	62	89%
6	Female	33	Falling down	88	56	90%
7	Male	22	Falling down	100	67	88%
8	Male	24	Falling down	90	60	91%
9	Female	29	Falling down	94	60	94%

The average duration of the surgical procedure was 63.78 ± 5.3 minutes, and an average of 94 intraoperative radiographic images were taken during the procedures, ranging from 85 to 103 images.

After a thorough clinical and radiological evaluation, the average time required for fractures to heal was 18 ± 2 weeks. Notably, no complications were observed during the surgeries or the follow-up period. Radiological assessments confirmed that the tibia nail was positioned satisfactorily.

**Figure 5:**
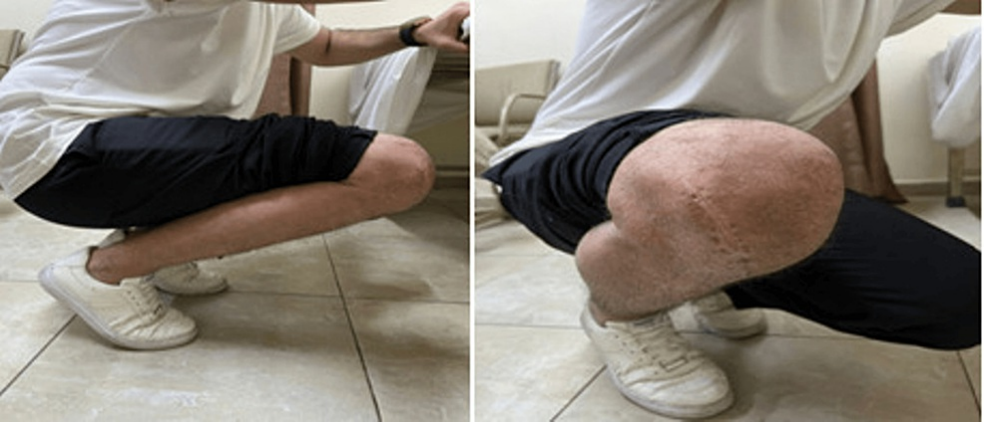
Gross photos taken at the clinic after six weeks showing full flexion, and the scar representing the lateral parapatellar approach.

At the final six-month follow-up, the mean Kujala score was 90.9% ± 2.3%, ranging from 88% to 94%. All patients regained a comparable range of motion in their knees and ankles. One patient reported minimal pain at the pin site but did not require any analgesia (Figure 5).

## Discussion

Tibia shaft fracture treatment with intramedullary nailing is a frequent procedure performed by orthopedic surgeons. Different surgical approaches for the entry point were described for tibia nailing. Appropriate positioning of the patient leads to a decrease in the operative time and radiation exposure during the surgery. Additionally, techniques that lower anterior knee pain improve the outcome and patient satisfaction.

Many studies compared infrapatellar and suprapatellar intramedullary nailing and concluded that the suprapatellar approach might decrease radiation exposure and allow more straightforward reduction, which leads to a shorter operative time. The suprapatellar approach is performed under a semi-extended position, which we believe is a key element affecting trials needed for reduction, hence the operative time and the need for fluoroscopic images. Moreover, the lateral parapatellar approach plays a role in the accessibility of the entry point without harming the surrounding soft tissue structures to avoid the possible causes of anterior knee pain [14,18,19].

Our study measured operative time, radiation exposure, and anterior knee pain in nine patients with tibial shaft fracture treated with lateral parapatellar intramedullary technique. We found comparable results to studies done on the suprapatellar tibia nailing technique. Williamson et al. and Franke et al. found that the semi-extended knee position used in suprapatellar nailing, which is very similar to the position in our study, aids in reducing and decreasing the fluoroscopic time [20,21].

As we mentioned, many papers compare the results between suprapatellar and infrapatellar approaches; however, why should we consider the lateral parapatellar approach over the suprapatellar approach?

The lateral parapatellar approach may provide the advantage of having more accessibility to the entry point with fewer complications, including less damage to the articular surface during the reaming, including the patellofemoral joint. Therefore, this may contribute to reducing anterior knee pain. Additionally, in their cadaveric study, Patel et al. demonstrated that using the parapatellar approach may not compromise joint capsule integrity reaching the entry point without avoiding the capsule, which was not done in our case series [22].

The suprapatellar approach requires specific suprapatellar nail design and equipment, while the parapatellar approach utilizes the same equipment as the infrapatellar approach.

Other studies recommended a medial parapatellar approach for tibia intramedullary nailing as an option. Nevertheless, this may contribute to higher lateral patellar instability risk.

Launder et al. described a similar approach to ours [14]. However, in our series, we made the incision more proximal, attempting to be more comfortable with the entry point and avoiding the tightening of the surrounding soft tissue during insertion. We preserved the rim of the lateral retinaculum to facilitate the closure and fasten the healing. Additionally, we avoided sharp dissection in the lateral retinaculum.

Positioning of the patient is a crucial point, and it affects the surgery progression significantly. The semi-extended position led to fewer trials of closed reductions and easy accessibility to the entry point, which will decrease the operation time and radiation exposure. The database of our center was reviewed, and it showed that the average fluoroscopic images for tibia nailing using the infrapatellar approach was 220, and the operation time average was 85 minutes, which is similar to the results by Rashid et al. [23,24]. In our case series, the average surgery duration was 63.78 ± 5.3 minutes, and the average intraoperative radiograph was 94 images, ranging from 85 to 105 images. 

In our series, we recommend the lateral parapatellar approach as it allows us to displace the patella and feel the entry point. This may increase accuracy and decrease the need for intraoperative radiographs and operative time. It did not compromise patellar instability and was associated with lower anterior knee pain. Furthermore, this study has potential limitations represented by its small sample size, relatively short follow-up duration, and the lack of accurate radiation dose measures.

## Conclusions

The lateral parapatellar approach for tibial shaft fracture nailing has the advantage of reducing operative time, the number of intraoperative radiographs, and lower postoperative anterior knee pain. Additionally, this approach did not cause patellar instability.

We recommend designing randomized controlled studies in the future comparing different techniques of tibia nailing with larger samples and studying its effects on the outcomes that we discussed in this study: operative time, radiation exposure, and anterior knee pain.
